# Creutzfeldt–Jakob disease: A case report

**DOI:** 10.1016/j.radcr.2024.11.011

**Published:** 2024-11-29

**Authors:** Fahad Rasool Butt, Thanansayan Dhivagaran, Syed Naqvi

**Affiliations:** a Schulich School of Medicine and Dentistry, University of Western Ontario, London, Ontario, Canada; b Department of Medical Imaging, Western University, Windsor, Ontario, Canada

**Keywords:** Creutzfeldt–Jakob disease, MRI, Neurodegenerative disorder, Spongiform encephalopathy, Prion disease, Rapidly progressive dementia

## Abstract

Creutzfeldt–Jakob Disease (CJD) is a rare, fatal neurodegenerative disorder that is caused by prion proteins. Patients often present with rapidly progressive dementia, ataxia, myoclonus, memory impairment, visual problems, and changes in personality. In this case report, we aimed to address the course of a 62 year old female who presented with progressive decline in cognitive function and died within 6 months of presentation. The patient underwent cerebrospinal fluid testing, MRI brain, and electroencephalography during her stay in the hospital. Ultimately, an autopsy was performed, which demonstrated spongiform changes, neuronal loss, and astrogliosis, consistent with CJD.

## Introduction

Creutzfeldt–Jakob Disease (CJD) is a fatal neurodegenerative disorder that is caused by prion proteins and falls under the group of conditions known as transmissible spongiform encephalopathies [1]. It occurs due to the accumulation of structurally abnormal prion proteins that are resistant to degradation and eventually spread through the brain [2]. This leads to the formation of a toxic aggregate which causes spongiform degeneration of brain tissue, neuronal loss, and brain tissue scarring [2]. CJD patients present with rapidly progressive dementia, ataxia, myoclonus, memory impairment, and visual problems, and die within the first year after symptom onset [3].

There are 3 kinds of CJD: sporadic, hereditary, and acquired (iatrogenic and variant). Sporadic CJD (sCJD) is the most common type of human prion disease accounting for approximately 85% of cases [4]. sCJD has an annual incidence of approximately 2 cases per million worldwide, with it rarely affecting the youth and having a greater mortality rate within the 60-79 age group [4]. The diagnosis of sCJD is based on presentation of clinical symptoms, utilization of electroencephalography of brain activity, MRI for detailed brain imaging, and cerebrospinal fluid testing for protein level detection [5]. To date, CJD has no cure, while there are drugs being studied for its treatment, current practice lies with management of symptoms and provision of palliative care [6].

## Case report

A 62 year old female presented to ER with progressive decline in cognitive function over the past 3 months, including ataxia, memory loss, and disorientation. During her hospital visit, CT of the head and chest/abdomen/pelvis were performed initially, which were negative for malignancy, infarction or hemorrhage. Multiple tests were performed to exclude causes such as infection, toxic or metabolic diseases, and autoimmune encephalitis.

MRI of the head was performed which demonstrated diffusion restriction and T2/FLAIR hyperintensities in the bilateral basal ganglia (caudate and putamen), bilateral thalami, hippocampi, and bilateral frontal and temporal cortices in a gyriform pattern. No corresponding enhancement or mass effect was seen. Lumbar puncture was performed, which demonstrated elevated levels of 14-3-3 protein in the cerebrospinal fluid. EEG was performed, which showed slowing activity and periodic sharp wave complexes (PSWC). Overall, the results of head MRI, EEG, and CSF testing were consistent with CJD.

**Fig. 1:**
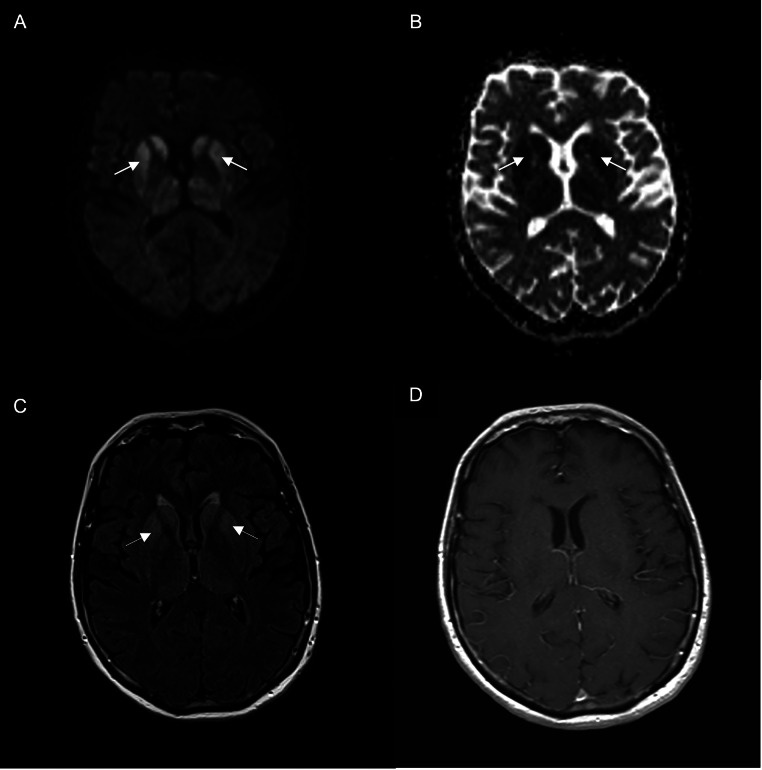
Axial DWI (A) and ADC (B) showed symmetric restricted diffusion in the bilateral caudate, putamen and thalami (thick arrows). Axial FLAIR (C) showed corresponding FLAIR hyperintensity (thin arrows). Axial contrast-enhanced T1 weighted sequence (D) showed no contrast enhancement.

Within a few weeks of admission, the patient had significant worsening of cognitive function and loss of speech output, and passed away peacefully with family at bedside. An autopsy was performed which demonstrated spongiform changes, neuronal loss, and astrogliosis, consistent with CJD Fig. 1.

## Discussion

CJD is a rare fatal neurologic condition generated by prion proteins. There are 3 kinds of CJD: sporadic, hereditary, and acquired (iatrogenic and variant) [1]. The most prevalent variety is sporadic, which happens when there are no risk factors present [1]. Genetic Creutzfeldt–Jakob disease develops by mutations in the PRNP gene [1]. Iatrogenic Creutzfeldt–Jakob disease is caused by exposure to prion-contaminated neurosurgical instruments, often in relation to medical or surgical procedures [1,2]. Variant Creutzfeldt–Jakob disease is caused by absorbing prion-contaminated beef products [1].

CJD manifests with a range of clinical symptoms, mostly marked by rapidly advancing dementia [7]. Patients often experience a rapid deterioration in cognitive function, memory impairment, impaired visual acuity or perceptual distortions, cerebellar dysfunction resulting in impaired coordination and equilibrium, and extrapyramidal symptoms characterized by muscle stiffness and reduced ability to initiate movement [7]. As the illness advances, individuals with CJD often have worsening neurological impairments, such as akinetic mutism, characterized by immobility and an inability to speak [8]. The illness progresses swiftly, usually resulting in profound disability and death within 1 year of onset [8].

Diagnosis of CJD can be challenging during its early stages due to its rarity and overlap of symptoms with other diseases [9]. Differential considerations of progressive dementia include many entities such as Alzheimher's disease, Lewy body dementia, frontotemporal dementia, meningoencephalitis, corticobasal degeneration, progressive supranuclear palsy, CADASIL, and paraneoplastic encephalitis [9].

According to the Centers for Disease Control and Prevention (CDC) in 2018, a definite diagnosis of CJD can only be established through positive brain tissue pathology [10]. Probable diagnosis can be made if the patient presents with rapidly progressive dementia accompanied by at least two of the following: myoclonus, visual and/or cerebellar signs, pyramidal and/or extrapyramidal signs, or akinetic mutism [10]. Probable diagnosis also requires positive findings on one of the brain MRI, EEG or CSF testing [10].

Brain MRI is an important diagnostic tool as it is relatively noninvasive and demonstrates high sensitivity in detection for CJD. The sensitivity of MRI in the detection of CJD have been reported around 90% [[11], [12], [13]]. Its specificity is limited due to overlap of imaging findings with other neurologic disorders such as hypoxic ischemic encephalopathy, encephalitis, and metabolic disorders such as hepatic encephalopathy or hypoglycemia [14]. In the case of CJD, there is typically diffusion restriction and T2/FLAIR hyperintensity involving the cortex and the basal ganglia (caudate and putamen) [14]. Cortical involvement can be focal or diffuse and symmetric or asymmetric [14]. Thalamic involvement (unilateral or bilateral) has been reported with variant CJD, but can be seen with sporadic CJD as well [14]. Thalamic hyperintense signal abnormalities are typically in the pulvinar and dorsomedial thalamic nuclei, also known as the “hockey stick sign” [14]. As per CDC diagnostic criteria, positive MRI findings are considered to be DWI/FLAIR hyperintense signal in the caudate/putamen or in at least 2 cortical regions (temporal, parietal, occipital lobes) [15]. DWI sequence is reported as being more sensitive when compared to T2 and FLAIR sequences for early detection [16].

The EEG in patients with CJD will show characteristic changes based on the progression of the disease [17]. At an early stage, there may be nonspecific findings such as generalized slowing and frontal rhythmic delta activity. As the disease progresses to middle and late stages, the EEG commonly shows periodic sharp wave complexes (PSWCs) [17].

In addition to EEG findings, CSF testing also has an important role in the diagnosis of CJD. CSF testing involves the detection of 14-3-3, S100B, and total Tau proteins, as well as a real-time quaking induced conversion (RT-QuIC) test [18]. The 14-3-3, S100B, and total tau proteins are biomarkers that can help assess the probability of prion disease [18]. However, these biomarkers are nonspecific and can be increased due to other conditions like Alzheimer's disease, CNS infections, stroke, etc. [18]. Including a RT-QuIC test can greatly aid in the diagnosis of prion disease as it has a 90.3% sensitivity rate and 98.5% specificity rate among patients who are screened for suspected prion disease [19]. Typically, there are high levels of these proteins in patients with sCJD, while sensitivity is worse for other types of CJD [20].

At present, there is no known treatment for CJD [21]. The treatment focuses on symptom management and palliative care [21]. However, research is being conducted on various potential therapies for CJD, such as the use of monoclonal antibodies, immunotherapy, and antiviral agents to stabilize or prevent the formation of abnormal prion proteins [21].

## Conclusion

In conclusion, CJD is a rare, fatal neurodegenerative disorder that is caused by prion proteins. This case report highlights the multifaceted nature of diagnosing CJD. Its diagnosis requires the integration of clinical presentation of rapidly progressive dementia with brain MRI results, cerebrospinal fluid analysis, and EEG testing. Although there is no known cure for CJD yet, novel diagnostic and therapeutic strategies are being explored to better address the challenges posed by this devastating disease.

## Patient consent

The patient's legal guardian provided written informed consent.

## References

[1] Sitammagari KK, Masood W. (2024). StatPearls.

[2] Vacca VM (2016). CJD: understanding Creutzfeldt-Jakob disease. Nursing 2023.

[3] Uttley L, Carroll C, Wong R, Hilton DA, Stevenson M. (2020). Creutzfeldt-Jakob disease: a systematic review of global incidence, prevalence, infectivity, and incubation. Lancet Infect Dis.

[4] Zerr I, Parchi P. (2018).

[5] Bahl JM, Heegaard NH, Falkenhorst G, Laursen H, Høgenhaven H, Mølbak K (2009). The diagnostic efficiency of biomarkers in sporadic Creutzfeldt-Jakob disease compared to Alzheimer's disease. Neurobiol Aging.

[6] Miranda LH, Oliveira AF, Carvalho DM, Souza GM, Magalhães JG, Cabral JA (2022). Systematic review of pharmacological management in Creutzfeldt-Jakob disease: no options so far?. Arquivos de Neuro-Psiquiatria.

[7] Mayo Clinic (2023). https://www.mayoclinic.org/diseases-conditions/creutzfeldt-jakob-disease/symptoms-causes/syc-20371226.

[8] Alzheimer's Association (2023). https://www.alz.org/alzheimers-dementia/what-is-dementia/types-of-dementia/creutzfeldt-jakob-disease.

[9] Kojima G, Tatsuno BK, Inaba M, Velligas S, Masaki K, Liow KK. (2013). Creutzfeldt-Jakob disease: a case report and differential diagnoses. Hawaii J Med Public Health.

[10] Centers for Disease Control and Prevention (2020). https://www.cdc.gov/creutzfeldt-jakob/hcp/clinical-overview/diagnosis.html.

[11] Valente AP, Pinho PD, Lucato LT. (2015). Magnetic ressonance imaging in the diagnosis of Creutzfeldt-Jakob disease: report of two cases. Dement Neuropsychol.

[12] Shiga Y, Miyazawa K, Sato S, Fukushima R, Shibuya S, Sato Y (2004). Diffusion-weighted MRI abnormalities as an early diagnostic marker for Creutzfeldt–Jakob disease. Neurology.

[13] Young GS, Geschwind MD, Fischbein NJ, Martindale JL, Henry RG, Liu S (2005). Diffusion-weighted and fluid-attenuated inversion recovery imaging in Creutzfeldt-Jakob disease: high sensitivity and specificity for diagnosis. Am J Neuroradiol.

[14] Prodi E, Rossi S, Bertaina I, Pravatà E, Sacco L. (2020). Report of a case of Creutzfeldt-Jakob disease with an unusual clinical presentation. Front Behav Neurosci.

[15] Zerr I, Kallenberg K, Summers DM, Romero C, Taratuto A, Heinemann U (2009). Updated clinical diagnostic criteria for sporadic Creutzfeldt-Jakob disease. Brain.

[16] Tschampa HJ, Kallenberg K, Urbach H, Meissner B, Nicolay C, Kretzschmar HA (2005). MRI in the diagnosis of sporadic Creutzfeldt–Jakob disease: a study on inter-observer agreement. Brain.

[17] Wieser HG, Schindler K, Zumsteg D. (2006). EEG in Creutzfeldt–Jakob disease. Clin Neurophysiol.

[18] Coulthart MB, Jansen GH, Cashman NR. (2014). Interpretation of cerebrospinal fluid protein tests in the diagnosis of sporadic Creutzfeldt–Jakob disease: an evidence-based approach. CMAJ.

[19] Rhoads DD, Wrona A, Foutz A, Blevins J, Glisic K, Person M (2020). Diagnosis of prion diseases by RT-QuIC results in improved surveillance. Neurology.

[20] Sanchez-Juan P, Green A, Ladogana A, Cuadrado-Corrales N, Sáanchez-Valle R, Mitrováa E (2006). CSF tests in the differential diagnosis of Creutzfeldt-Jakob disease. Neurology.

[21] Mead S, Khalili-Shirazi A, Potter C, Mok T, Nihat A, Hyare H (2022). Prion protein monoclonal antibody (PRN100) therapy for Creutzfeldt–Jakob disease: evaluation of a first-in-human treatment programme. Lancet Neurol.

